# Optimized methods for extracting circulating small RNAs from long-term stored equine samples

**DOI:** 10.1186/s13028-016-0224-5

**Published:** 2016-06-29

**Authors:** Lucia Unger, Nathalie Fouché, Tosso Leeb, Vincent Gerber, Alicja Pacholewska

**Affiliations:** 1 Department of Clinical Veterinary Medicine, Swiss Institute of Equine Medicine, Vetsuisse Faculty, University of Bern and Agroscope, Länggassstrasse 124, 3012 Bern, Switzerland; 2 Department of Clinical Research and Veterinary Public Health, Institute of Genetics, Vetsuisse Faculty, University of Bern, Bremgartenstrasse 109A, 3012 Bern, Switzerland

**Keywords:** Horse, Small RNA, microRNA, miRNA, RNA extraction, Serum, EDTA

## Abstract

Circulating miRNAs in body fluids, particularly serum, are promising candidates for future routine biomarker profiling in various pathologic conditions in human and veterinary medicine. However, reliable standardized methods for miRNA extraction from equine serum and fresh or archived whole blood are sorely lacking. We systematically compared various miRNA extraction methods from serum and whole blood after short and long-term storage without addition of RNA stabilizing additives prior to freezing. Time of storage at room temperature prior to freezing did not affect miRNA quality in serum. Furthermore, we showed that miRNA of NGS-sufficient quality can be recovered from blood samples after >10 years of storage at −80 °C. This allows retrospective analyses of miRNAs from archived samples.

**Electronic supplementary material:**

The online version of this article (doi:10.1186/s13028-016-0224-5) contains supplementary material, which is available to authorized users.

## Findings

Non-coding microRNAs (miRNAs), amongst other small RNAs, play an important role in shaping a cell’s transcriptome profile by regulation of mRNA transcription and/or translation [1] and may thus affect multiple target genes. In human medicine, miRNAs are promising diagnostic and prognostic biomarkers in cancer and other complex diseases [2].

In equine medicine, the knowledge about miRNAs in physiologic and pathologic conditions is still very limited restricted to certain diseases only, and thus warrants further investigation in this species [3–6].

In contrast to mRNA, miRNA molecules are surprisingly stable—even in tissue samples of compromised quality [7]—which supports the possibility of routine use of miRNA fingerprints as biomarkers in clinical practice. Small RNA extraction methods have been established for model organisms like human and mouse. Still, optimized procedures for circulating miRNA extraction might differ among species and there is little experience with extraction from equine samples [8, 9]. In serum and plasma, extracellular miRNAs are only present in low concentrations and their exact measurements may be affected by interference with DNA, degraded RNA, erythrocyte-derived miRNAs, and blood contaminants like heme and immunoglobulins [10, 11].

For miRNA studies using equine EDTA blood, the use of PAXgene blood RNA tubes in combination with the PAXgene blood RNA extraction kit was recommended [6]. However, particularly for retrospective studies, archived blood samples stored for years without any RNA stabilizing additives may be the only available material. The aim of our study was to compare different methods for the isolation of miRNAs from equine serum, fresh and archived EDTA blood samples.

For miRNA extraction from serum samples we assessed how RNA yield is affected by the performance of three column-based and one column-free method, two different lysis reagents, and the effects of storage time at room temperature (RT), serum input volume, performing a second phenol extraction, and a second column elution. We performed our analysis on serum samples from three horses. As we assessed a large number of different parameters, we did not analyse all possible 576 combinations, but rather chose an incomplete matrix of 60 combinations that allowed us to address each of the six investigated parameters (Additional file 1).

RNA isolation was performed according to the protocols of the four different manufacturers: ZR Whole-Blood RNA MiniPrep, Zymo Reasearch; Direct-zol RNA MiniPrep, Zymo Research; miRNeasy Serum/Plasma Kit, Qiagen), and standard TRIzol method [12]. The kits resulted in comparable miRNA yields that ranged from 0.13 to 0.64 ng RNA/100 μl serum (Table 1). The Direct-zol RNA MiniPrep was the fastest procedure, however, it had the lowest maximum sample loading volume (up to 100 μl). The highest final concentrations of the isolated small RNA were obtained with the miRNeasy and TRIzol methods (up to 138 pg/μl, Table 1). We chose the miRNeasy serum/plasma kit as our preferred method as the column-based purification is less prone to handling errors. The use of TRIzol as lysis buffer in combination with miRNeasy columns revealed similar results in terms of RNA yield as QIAzol, which is recommended by the manufacturer (Wilcoxon signed rank test *P* = 0.57).

**Table 1:** Small RNA extraction kit comparison

Kit	Horse ID	Serum volume (μl)	RNA concentration, Nanodrop (ng/μl)	Elution volume (μl)	Small RNA concentration, Bioanalyzer (pg/μl)	miRNA in small RNA (%)	miRNA/100 μl serum (ng)
ZR WB	1	200	1.85	8	89.4	46	0.17
ZR WB	3	200	3.65	8	69.5	63	0.18
Direct-zol	1	100	2.70	50	15.0	86	0.64
Direct-zol	3	100	0.45	50	12.8	53	0.34
miRNeasy	1	200	56.23	12	30.4	72	0.13
miRNeasy	3	200	11.69	12	126.2	77	0.58
TRIzol	1	400	52.98	10	138.5	63	0.22
TRIzol	3	400	138.21	10	127.9	43	0.14

RNA extraction results with different kits used. Due to differences in serum and elution volumes used, the RNA/miRNA concentration is given in RNA per 100μl of serum. RNA was measured with Nanodrop and miRNA with Bioanalyzer. The concentration was not measurable with the Qubit high sensitivity RNA kit. Due to very low concentration, the Nanodrop results should be treated with caution

We next assessed the effect of serum storage times on the performance of the miRNeasy method (Additional File 1). Serum aliquots were stored for 1, 5, or 24 h at RT before freezing at −80 °C. The storage time at RT did not have a significant effect on the RNA yield per 100 μl serum used (Kruskal–Wallis test *P* = 0.12) (Additional file 1).

To increase the efficiency of the RNA extraction with miRNeasy serum/plasma kit the following procedures were tested: (1) second extraction: by adding 600 μl RNase-free water to the remaining organic phase, and further steps as indicated by the manufacturer; (2) second elution: re-using the first eluate; and (3) increased serum volume: 5× or 10× multiplication of 200 μl serum processed individually for the phase separation and then loaded sequentially on a single column (Additional file 1). The second elution did not show a significant increase in RNA yield (Wilcoxon signed rank test *P* = 0.07) and after the second extraction RNA was no longer detectable and therefore none of those steps were considered as advantageous. Increased serum volume improved the RNA yield in samples processed with QIAzol, therefore, 1 ml of serum was chosen as the minimum and 2 ml as the optimum volume balancing RNA yield and the time required for the procedure.

**Fig. 1:**
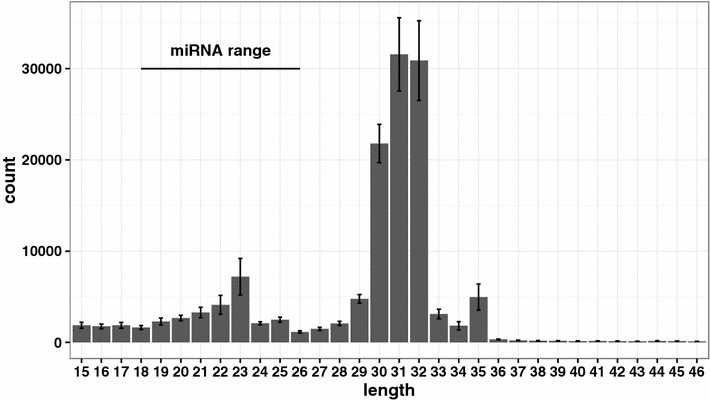
Sequencing tag length distribution. The reads length distribution of six serum small RNA libraries is shown. The *bars* indicate mean number of tags per sample and the error bars indicate standard errors

To further assess the quality of the small RNA extracted with the optimised procedure we used six serum small RNA samples (36–174 ng) for next generation sequencing on a MiSeq (Additional file 1). Pre-processed sequencing reads in FASTQ format are available at http://www.ebi.ac.uk/ena/data/view/PRJEB10829. Read length distribution revealed a peak within the miRNA range and an even higher peak within the piRNA range (Fig. 1). This was in agreement with a previous study performed on pig serum [9].

To corroborate our findings we isolated 120 miRNA samples with the optimized protocol (Additional file 2). The RNA yield ranged from 4 to 876 ng/100 μl serum (mean = 39 ng/100 μl serum). Only two samples showed high level of haemolysis and therefore the effect of haemolysis on serum RNA content [13] could not be investigated in this study.

**Fig. 2:**
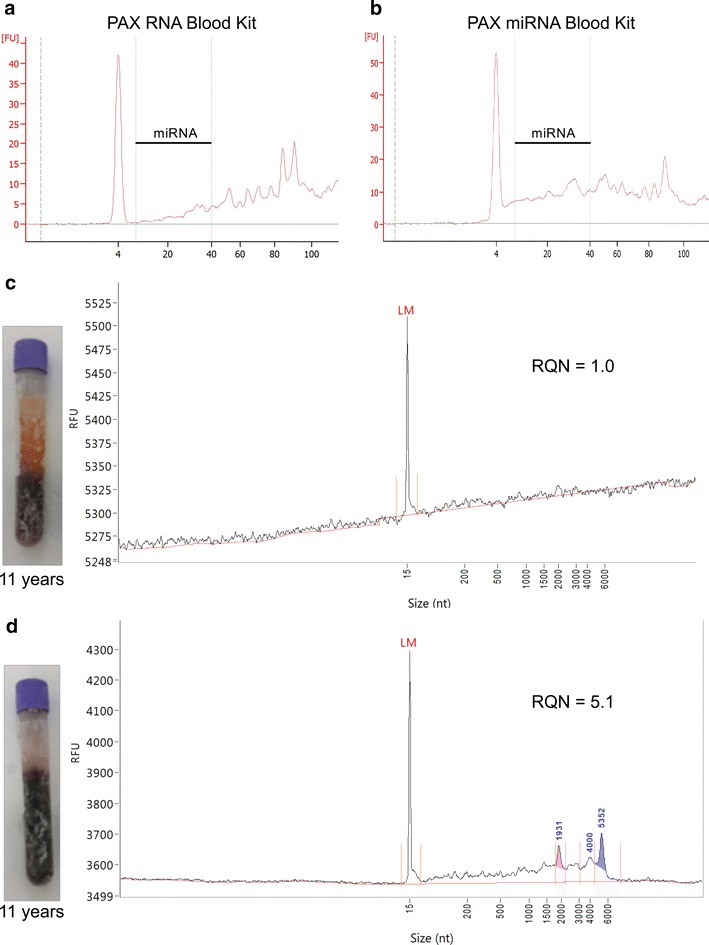
RNA quality of equine EDTA blood samples. Amount of miRNA (Bioanalyzer, small RNA chip) derived from the same short-term stored sample extracted with PAXgene blood RNA (**a**) or miRNA (**b**) kit. RNA quality (Fragment Analyzer) of long-term stored samples (11 years) with (**c**) or without (**d**) phase separation extracted with PAXgene blood RNA kit

For the optimization of miRNA extraction from archived EDTA blood samples we compared the PAXgene blood RNA kit with the PAXgene blood miRNA kit that is designed specifically for small RNA enrichment. For this comparison we used six horse EDTA samples stored for 12 weeks, 5 or 11 years (Additional file 3) at −80 °C without addition of RNA stabilizing agents. Before thawing, samples were visually inspected for separation of plasma and red blood cells which was considered to be a sign of prolonged storage at RT prior to freezing. All samples were gently thawed on ice and 2.5 ml was transferred to a PAXgene blood RNA tube and incubated for 16 h as previously described [14]. Samples extracted with both kits did not show significantly different RNA concentration (Wilcoxon signed rank test *P* = 0.16) whereas the miRNA in small RNA ratio (Agilent 2100 Bioanalyzer) was increased if the PAXgene blood miRNA kit was used (Fig. 2a, b).

We next assessed the effect of (1) storage time and (2) sample handling before freezing on the RNA quality and quantity using the PAXgene blood miRNA kit and EDTA blood samples derived from 25 horses (Additional file 3). Samples of two different sampling periods were compared (11 short-term stored samples: 12 weeks vs. 14 long-term stored samples: 5–11 years): Whereas no significant changes in RNA yield could be identified between samples of the two sampling periods (Wilcoxon rank sum test *P* = 0.18), the RNA quality was significantly lower in long-term stored samples (Wilcoxon rank sum test *P* = 6.10e−4). Phase separation in long-term stored samples did not have a significant effect on RNA concentration (Wilcoxon rank sum test *P* = 0.31), but had a significant effect on RNA quality (Wilcoxon rank sum test *P* = 1.02e−4) (Fig. 2c, d). In archived samples without visible phase separation (n = 5), the best RNA quality achieved was RQN = 8.3 (sample stored 9 years, Additional file 3).

All 120 serum and 21 EDTA small RNA samples extracted with optimized procedures were successfully converted into single-end libraries and sequenced on an Illumina HiSeq (Additional file 2). The number of sequencing tags after data pre-processing ranged from 7.1 to 16.5 million.

In this study we showed that compared to human an increased volume of equine serum is needed to obtain sufficient amounts of small RNA for sequencing, as in bovine samples [9]. However, using manual extraction only 2 ml of serum is needed, compared to 9 ml for the previously described extraction with the QIAcube robotic system [9]. Similar to [14], we were able to rescue RNA of good quality from some long-term stored equine EDTA blood samples via gentle thawing on ice and transfer to a PAXgene blood RNA tube.

## Additional files

**Additional file 1.** RNA extraction from serum. A table in XLSX format with RNA concentration and yield measured with Qubit, miRNA concentration and rRNA contamination measured with Bioanalyzer in samples used for pilot experiments during method optimization. Read numbers of the six samples sequenced are also given.

**Additional file 2.** Serum RNA extraction results from 120 samples. A table in XLSX format with serum absorbances at 414 nm, serum volume used, sample collection year, and RNA concentrations obtained using the optimized procedures and measured with Qubit and Bioanalyzer.

**Additional file 3.** RNA extraction from EDTA. A table in XLSX format with RNA concentration and yield measured with Qubit, miRNA concentration measured with Bioanalyzer, and RNA quality number (RQN) measured by Fragment Analyzer in samples used for pilot experiments during method optimization and with already optimized method.

## Abbreviations

EDTA: ethylenediamine tetraacetic acid
miRNA: micro RNA
RT: room temperature, i.e. around 20 °C
